# Editorial: Computer vision and image synthesis for neurological applications

**DOI:** 10.3389/fncom.2025.1561635

**Published:** 2025-02-10

**Authors:** Chenglong Zou, Shounak Roychowdhury, Saim Rasheed, Raza Ali

**Affiliations:** ^1^School of Electronics and Internet of Things, Chongqing Polytechnic University of Electronic Technology, Chongqing, China; ^2^Peking University Chongqing Research Institute of Big Data, Chongqing, China; ^3^School of Mathematical Science, Peking University, Beijing, China; ^4^The University of Texas at Austin, Austin, TX, United States; ^5^King Abdulaziz University, Jeddah, Saudi Arabia; ^6^Balochistan University of Information Technology, Quetta, Pakistan

**Keywords:** computer vision, image synthesis, neurological disorders, disease diagnosis, deep learning - artificial intelligence

Computer vision and image synthesis based on deep learning models, such as YOLO, U-Net, and Transformer, are advancing rapidly. These technologies are significantly impacting the field of neurology. They offer more precise, expedient, and reliable techniques for diagnosing, treating, and monitoring neurological disorders. Based on this observation, we initiated a latest article collection on this Research Topic and received a total of 8 submissions. Finally, 4 distinguished papers are accepted for publication after the considerable and rigorous peer reviews. We would like to express our sincere thanks and congratulations to all authors and reviewers. More importantly, the following contributions and highlights are worth learning for relevant researchers.

## Paper 1: An efficient computer vision-based approach for acute lymphoblastic leukemia prediction

This paper (Almadhor et al.) employs machine learning techniques to select relevant features from a large-size feature set acquired and a pre-trained Convolutional Neural Networks (CNNs) are used for feature extraction and classification. The proposed approach is helpful for medical experts and patients by diagnosing acute lymphocytic leukemia early, which could significantly improve patient outcomes.

## Paper 2: EEG-based emotion recognition using graph convolutional neural network with dual attention mechanism

This paper (Chen W. et al.) utilizes graph convolutional neural networks to model the brain network as a graph to extract representative spatiotemporal features. Besides, two attention mechanisms are used: electrode channel attention and signal frequency band attention. These mechanisms provide insights into how different brain regions and signal frequency bands contribute to emotion generation.

## Paper 3: Enhancing brain tumor detection in MRI images using YOLO-NeuroBoost model

This paper (Chen A. et al.) introduces the YOLO-NeuroBoost model, which integrates multiple innovative technologies to address the accuracy and efficiency limitations of existing methods in brain tumor detection. The methodology demonstrates strong practicality and versatility, not only for brain tumor detection but also potentially for other medical imaging applications.

## Paper 4: MUNet: a novel framework for accurate brain tumor segmentation combining UNet and mamba networks

This paper (Yang et al.) proposes MUNet, a novel network framework that combines the advantages of UNet and Mamba for brain tumor segmentation. A special SSM-based structure called the SD-SSM Block and the SD-Conv structure, enhances segmentation performance by capturing multi-scale global and local features and compressing redundant information between features. Besides, they use a novel loss function that combines mIoU, Dice, and Boundary losses to optimize the segmentation's overlap and similarity. These innovations improve the accuracy and efficiency of brain tumor segmentation.

Overall, the contributions and work presented in these papers span various aspects of medical imaging and machine learning. Paper 1 focuses on feature selection and classification for early medical diagnosis. Paper 2 proposes a dual attention mechanism graph convolutional neural network for emotion generation based on EEG. Paper 3 introduces the YOLO-NeuroBoost model for enhancing brain tumor detection. Finally, Paper 4 presents MUNet, a novel network framework for brain tumor segmentation. Each paper provides significant advancements in their respective fields, demonstrating the potential of machine learning and deep learning techniques in medical imaging applications.

